# Assessment of the Live Attenuated and Wild-Type *Edwardsiella ictaluri*-Induced Immune Gene Expression and Langerhans-Like Cell Profiles in the Immune-Related Organs of Catfish

**DOI:** 10.3389/fimmu.2019.00392

**Published:** 2019-03-06

**Authors:** Adef O. Kordon, Hossam Abdelhamed, Hamada Ahmed, Wes Baumgartner, Attila Karsi, Lesya M. Pinchuk

**Affiliations:** ^1^Department of Basic Sciences, College of Veterinary Medicine, Mississippi State University, Mississippi State, MS, United States; ^2^Department of Nutrition and Veterinary Clinical Nutrition, Faculty of Veterinary Medicine, Damanhour University, Damanhour, Egypt; ^3^Department of Pathobiology and Population Medicine, College of Veterinary Medicine, Mississippi State University, Mississippi State, MS, United States

**Keywords:** Langerhans cells, channel catfish, *Edwardsiella ictaluri*, live attenuated vaccines, cytokines/chemokines, antigen presentation

## Abstract

*Edwardsiella ictaluri* is a Gram-negative intracellular pathogen that causes enteric septicemia of catfish (ESC). Successful vaccination against intracellular pathogens requires T cell priming by antigen presenting cells (APCs) that bridge innate and adaptive immunity. However, the evidence on immunological mechanisms that underscore *E. ictaluri* pathogenesis and the protective role of live attenuated vaccines (LAVs) is scarce. We assessed the expression of immune genes related to antigen presentation by real-time PCR and the distribution patterns of Langerhans-like (L/CD207^+^) cells by immunohistochemistry in the immune-related tissues of channel catfish challenged with two novel *E. ictaluri* LAVs, *Ei*Δ*evpB*, and ESC-NDKL1 and wild type (WT) strain. Our results indicated significantly elevated expression of IFN-γ gene in the anterior kidney (AK) and spleen of vaccinated catfish at the early stages of exposure, which correlated with increased numbers of L/CD207^+^ cells. In general, the ESC-NDKL1-induced IFN-γ gene expression patterns in the AK resembled that of the patterns induced by *Ei*Δ*evpB*. However the MHCII gene expression patterns differed between the strains with significant increases at 6 h post-challenge (pc) with the *Ei*Δ*evpB* and at 7 d pc with the ESC-NDKL1 strains, respectively. Significant increases in activity of T helper type polarization genes such as IFN-γ and T cell co-receptors after exposure to ESC-NDKL1, in combination with elevated numbers of L/CD207^+^ cells at 7 d pc with both LAVs compared to uninfected and the WT-exposed counterparts, were documented in the spleen. The dominant pro-inflammatory environment with dramatically overexpressed inflammatory genes in the AK and 7 d pc in the spleen in response to *E. ictaluri* was found in exposed catfish. In general, the pro-inflammatory gene expression profiles in the ESC-NDKL1 pc showed more similarities to the WT strain-induced gene profiles compared to the *Ei*Δ*evpB* counterpart. In addition, *E. ictaluri* WT significantly decreased the numbers of Langerhans-like L/CD207^+^ cells in the AK and spleen at 3 and 7 days pc. In conclusion, we report the differential framework of initiation of innate and adaptive immune responses between *E. ictaluri* strains with both LAVs having a potential of satisfying the stringent requirements for successful vaccines.

## Introduction

*Edwardsiella ictaluri* (*E. ictaluri*) is a Gram-negative facultative intracellular pathogen that causes enteric septicemia of channel catfish (ESC), one of the most devastating diseases in the US catfish industry (1–4). A live *E. ictaluri* vaccine (Aquavac-ESC) against ESC was developed by Klesius and Shoemaker, and this vaccine can provide efficient protection to juvenile catfish (5). Then, immersion studies demonstrated that Aquavac-ESC stimulated the protective immunity in catfish fry, fingerlings, and eyed catfish eggs (6–9). Recently, avirulent *E. ictaluri* isolate (S97-773) was developed by Wise et al. and oral vaccination with this live attenuated isolate protected fingerlings from *E. ictaluri* infection (10). *Edwardsiella ictaluri* can survive and replicate in channel catfish macrophages, and *E. ictaluri* live attenuated vaccines (LAVs) induced cell-mediated immunity to protect catfish against ESC (11–13). Also, catfish vaccinated with LAVs triggered humoral immune responses which augmented the bacterial killing activity of macrophages (12, 14).

Our research group has developed two novel *E. ictaluri* LAV strains (*Ei*Δ*evpB* and ESC-NDKL1), which provided significant protection against ESC in both catfish fry and fingerlings (15, 16). *Ei*Δ*evpB* was constructed by in-frame deletion of the *evpB* gene, one of the main components of type six secretion system (T6SS) (15). ESC-NDKL1 (Δ*gcvP*Δ*sdhC*Δ*frdA*) was constructed by in-frame deletion of three genes in the tricarboxylic acid cycle (*sdhC* and *frdA*) and one-carbon metabolism (*gcvP*) (16). Although we know the genetic differences among the strains, their phenotypes have not been characterized. Laboratory and field challenge study demonstrated that *Ei*Δ*evpB* and ESC-NDKL1 are safe in catfish fingerlings and provide significant protection from disease. In 7–14 days old fry, *Ei*Δ*evpB* was found to be completely attenuated while ESC-NDKL1 showed 3–4% mortality (16). Recently, we demonstrated the phagocytic and killing properties of catfish peritoneal macrophages that are induced by LAVs. However, the intensity of *Ei*Δ*evpB* phagocytic uptake was significantly higher compared to the ESC-NDKL1 uptake in catfish peritoneal macrophages (14).

Fish anterior kidney (AK) possesses hemopoietic tissue responsible for production of all blood elements (17–20). Several studies demonstrated that the AK is the target organ at the early time of *E. ictaluri* infection. Leukocytes containing *E. ictaluri* were detected in the AK of channel catfish at 48 h post-challenge (pc), and *E. ictaluri* was detected in the posterior kidney at 15 min pc (21). In addition, the dispersion of bioluminescent *E. ictaluri* was observed in the AK of catfish fingerlings at 15 min after intraperitoneal injection (22).

The spleen in mammals is one of the secondary lymphoid organs in which antigen presentation occurs, and adaptive immune responses are activated (23). Similar to mammals, the spleen serves as a secondary lymphoid tissue in teleost fish (24). In the immersion-exposed channel catfish, bioluminescent *E. ictaluri* was detected in the abdominal area at 60–72 h pc (22). It was demonstrated that as *E. ictaluri* loads increased, disease and mortalities progressed more rapidly. For instance, at high doses (2.5 × 10^7^-2.5 × 10^8^ CFU) fish died in about 2 days, but at low doses (2.5 × 10^2^-2.5 × 10^3^ CFU) fish died in about 6 days. Dissection of catfish organs revealed that the AK and spleen had high *E. ictaluri* load, while *E. ictaluri* presence was also detected in the gills (22). In the tissue persistence study, both mutant and WT *E. ictaluri* strains demonstrated similar trends over time. However, the overall mean CFU per gram of AK of *E. ictaluri* WT from all time points was significantly higher than that of the mutant strain. Similar results have been obtained in the tissue persistence study with *Ei*Δ*evpB* and ESC-NDKL1 in catfish fry (unpublished observation).

Dendritic cells (DCs) are the most potent antigen-presenting cells (APCs) and are critical players in bridging and shaping all innate and adaptive immune responses in vertebrates (25). Recently, DCs have been characterized in several teleost fish based on their morphology and function. For example, DC-like cells with mammalian DC morphology and T-cell stimulatory capability have been described in zebrafish (26, 27). DCs have also been identified in rainbow trout, barramundi, and medaka based on their morphology, motility, phagocytic ability, and T-cell activation properties (28–30). A previous study reported the presence of the major co-stimulatory molecules (e.g., CD80/CD86, and CD83) in zebrafish (31). Furthermore, similarly to mammals, a recent study showed that the surface molecules of zebrafish DCs (CD80/86/83/CD209^+^) could promote CD4^+^ naïve T-cell stimulation (32). Dendritic cells in mammals have multiple subsets, and Langerhans cells (LCs) are the distinct subset of DCs. Langerhans cells are present in the epidermis, and this unique location provides LCs with early recognition of pathogens, foreign chemicals, and self-antigens (33). Langerhans cells can engulf antigens and migrate to the secondary lymphoid tissues to present the antigen to naïve T-cells, thus initiating adaptive immune responses (34). Langerhans cells are uniquely characterized by Birbeck granules, which are rod-shaped organelles consisting of a superimposed and zippered membrane (35). Langerin is a type II transmembrane C-type lectin, which is a specific marker for LCs and associated with the formation of Birbeck granules (36, 37). The antigen capture function of Langerin triggers the induction of Birbeck granules by allowing routing antigens into Birbeck granules, thus providing non-classical antigen processing pathway and cross-presentation (37, 38). Several studies have identified cells with mammalian LC morphology in teleost fish. In particular, Langerin/CD207^+^ (L/CD207^+^) cells have been described in the AK and spleen of Atlantic salmon and rainbow trout. Also, the same research group has observed DC-like cells containing Birbeck -like granules in the gills of Chinook salmon during *Loma salmonae* infection (39). Recently, our group identified L/CD207^+^ cells in the channel catfish AK, spleen, and gills by immunohistochemistry (IHC), and described cells that resembled mammalian LC DCs containing Birbeck-like granules in the spleen, anterior and posterior kidneys, and gills of channel catfish by transmission electron microscopy (40).

It was demonstrated that the transcriptional regulator of the Zinc finger family DC-SCRIPT is expressed in all subsets of DCs in humans and mice (41, 42). Due to the lack of DC-specific markers in fish, DC-SCRIPT was considered as one of the markers for the barramundi fish DCs (30). A recent study showed that DC-SCRIPT in combination with MHCII expression was significantly upregulated in the AK of barramundi at both, 6 h and 1 d post-injection with peptidoglycan (PTG) and lipopolysaccharide (LPS), but relative expression of DC-SCRIPT was low at 3 d and 7 d (30). Also, another study reported that TLR ligands, such as LPS and Pam3CSK4, activated hematopoietic progenitor cells and induced their differentiation into myeloid cells, such as macrophages and DCs, in the bone marrow and spleen of mammals (43).

The effects of efficacious *E. ictaluri* LAVs on innate and adaptive immune responses, and, specifically, on innate antigen presentation are still unexplored. Therefore, we aimed to evaluate the immune gene and L/CD207^+^ LC-like cells distribution patterns in the AK, spleen, and gills of catfish challenged with two LAV and WT strains of *E. ictaluri*.

## Materials and Methods

### Bacterial Strains

Wild-type (WT) *E. ictaluri* strain 93–146, *Ei*Δ*evpB*, and ESC-NDKL1 were cultured in brain heart infusion (BHI) agar or broth (Difco, Sparks, MD) at 30°C. When required, media were supplemented with colistin (Col: 12.5 mg/ml, Sigma-Aldrich, Saint Louis, MN).

### Catfish Vaccination and Tissue Collection

Two hundred specific-pathogen-free (SPF) channel catfish fingerlings (6-month old) were obtained from the College of Veterinary Medicine's fish hatchery at Mississippi State University, and all fish experiments were conducted according to the protocol approved by the Institutional Animal Care and Use Committee. Catfish were stocked into four 40-L tanks (25 fingerlings per tank) supplied with flow-through water and continuous aeration. Catfish were maintained at 25–28°C and fed twice daily with a floating catfish feed. After a week of acclimation, catfish were exposed to vaccine strains *Ei*Δ*evpB* and ESC-NDKL1, *E. ictaluri* WT (positive control), and BHI (sham-control) as previously described (44). Exposure dose was approximately 3.67 × 10^7^ CFU/ml of water, which was calculated by serial dilutions of bacterial cultures. At 6 h, 1, 3, and 7 pc, five catfish from each group were euthanatized by 300 mg/L tricaine methanesulfonate (Sigma, St. Louis, MO) and placed in 10% neutral buffered formalin for 48 h (formalin was replaced after 24 h with a fresh batch). For immunohistochemistry (IHC) analysis, anterior kidney (AK), spleen, and gill tissues were isolated, dehydrated with a graded series of ethanol, and embedded in paraffin wax. Finally, tissue sections were cut as described previously (45). For gene expression analysis, six more fish were euthanatized from each group at 6 h, 1, 3, 7, 14, and 21 d, and spleen and AK were collected and placed immediately into RNase-free tubes (ThermoFisher Scientific) that contained 10 volumes of RNA*later* (Ambion, Austin, TX).

### Immunohistochemistry (IHC)

**Figure 1:**
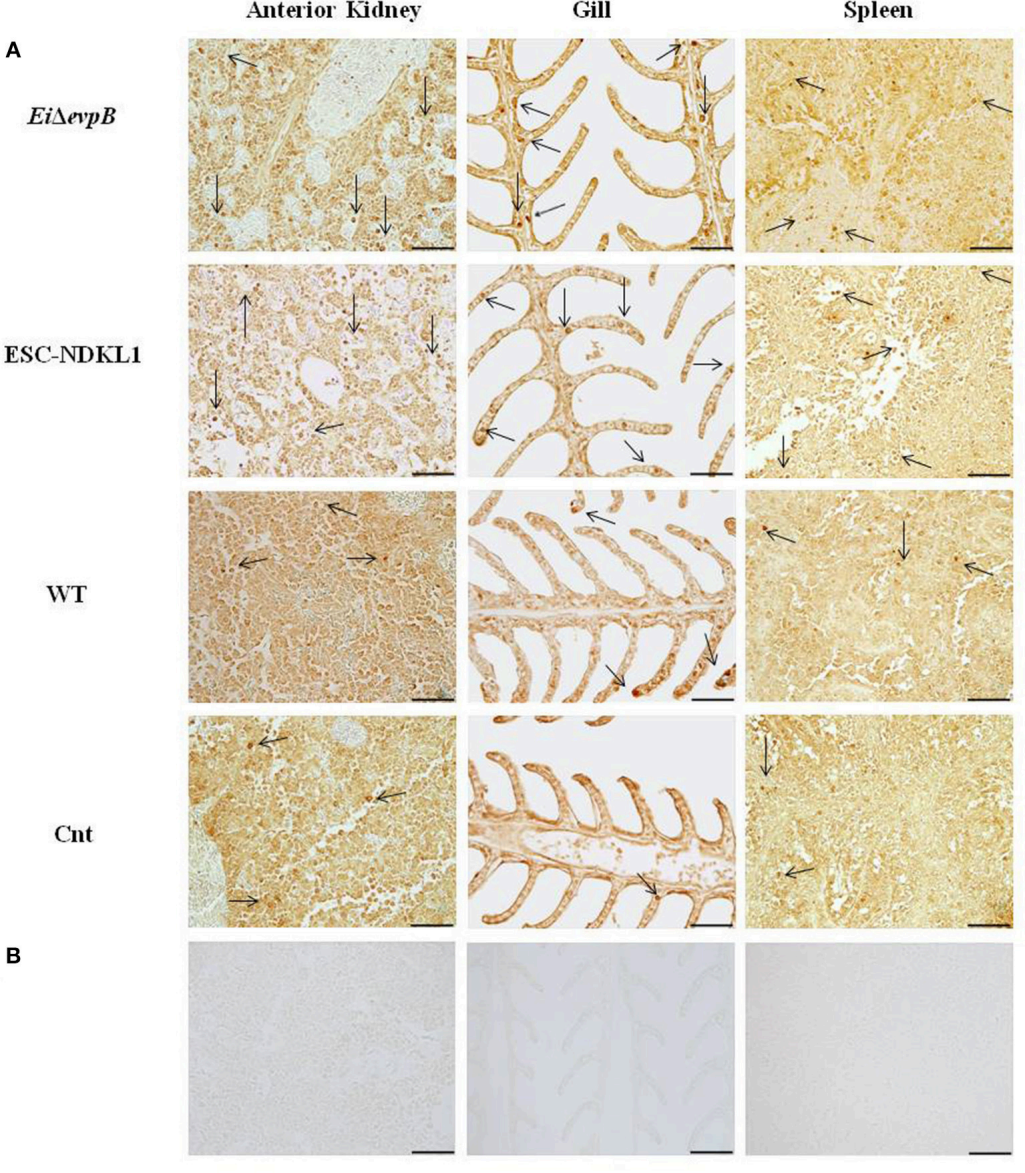
Identification of L/CD207^+^ cells by IHC in the lymphoid organs (AK, spleen) and gills of channel catfish challenged with *E. ictaluri* LAV and WT strains. The left column shows the L/CD207^+^ specific **(A)** and background staining **(B)** in AK of catfish in all groups at 3 d pc. Middle column indicates the L/CD207^+^ specific **(A)** and background staining **(B)** in the gill at 3 d pc. The right column shows the L/CD207^+^ specific **(A)** and background **(B)** staining in the spleen at 7 d pc. The arrows indicate L/CD207^+^ cells in the immune-related organs. Photomicrographs (400 x magnification, scale bar 50 mm).

To evaluate the numbers of L/CD207^+^ cells during *E. ictaluri* infection, immunohistochemical staining of catfish AK, spleen, and gill tissue was performed with polyclonal antibodies (pAbs) specific to human CD207 (R&D Systems, Inc.) as described previously (40). Briefly, for antigen retrieval, tissue sections were incubated in target retrieval solution (DAKO) for 40 min at 100°C. Then, tissue sections were allowed to cool at room temperature and were washed in 1X phosphate-buffered saline (PBS) for 10 min. Following washing, tissue sections were incubated in protein block (DAKO) for 1 h. After that, they were incubated with primary antibodies (0.2 mg/ml, 1:500 dilution). Normal goat IgG were used as negative controls at the same concentrations as primary antibodies. Primary and control antibodies were incubated in a humid chamber overnight, followed by the addition of streptavidin/biotin for 15 min and incubation with secondary antibodies (1:200 dilution, Biotinylated Anti-Goat IgG (H+L), Vector Laboratories) for 1 h. Following secondary antibody incubation, samples were incubated in Streptavidin-HRP (Vector Laboratories) for 1 h. Tissue sections were stained in a solution of 3,3′-diaminobenzidine tetrahydrochloride (DAB) for 10 min, and then washed twice with water for 10 min and dehydrated through a graded series of ethanol to xylene. Slides were analyzed at 40x magnification with an Olympus BX60 microscope (Olympus U-TV1 X) and photographed with Infinity analyze software (Lumenera corporation). The number of L/CD207^+^ cells in the AK, spleen, and gills of five catfish from each group was determined per each time point by using a “numbered indexed square grid” eyepiece graticule (1.00 mm^2^ IN DEX SQU, PYSER-SGILTD) (46). The L/CD207^+^ cell numbers were counted in 10 mm^2^ per field, and a total of 10 fields and 100 mm^2^ were counted for each organ. The mean value of L/CD207^+^ cells was calculated for further statistical analysis. The presence of the LC-like cells in the lymphoid organs and gills of catfish at different time points of pc is shown in Figure 1 and Supplementary Figures 1–3. In the following chapters, we provide detailed quantitative assessment of the numbers of L/CD207^+^ cells at 6 h, 1, 3, and 7 d post challenge that correlated with the kinetics of initiation of innate and adaptive immune responses in the immune-competent organs of catfish fingerlings.

### RNA Extraction and cDNA Synthesis

To isolate total RNA from the tissues, the FastRNA™ SPIN Kit for Microbes and the FastPrep-24™Instrument (MP Biomedicals, Santa Ana, CA) were used according to the manufacturer's instructions. DNase treatment with RNase-Free DNase Set (QIAGEN, Hilden, Germany) was used to eliminate any catfish gDNA contamination. The quantity and quality of total RNA were checked by using a NanoDrop ND-1000 spectrophotometer (Thermo Scientific, USA) and agarose gel.

We used Maxima First Strand cDNA Synthesis Kit for RT-qPCR (Thermo Scientific, USA) to convert total RNAs into cDNA according to the manufacturer's instructions. The cDNA was synthesized in a 20 μl reaction containing 2.5 ng of total RNA, 4 μl of 5X reaction mix, 2 μl of maximum enzyme mix, and nuclease-free water. The reactions were incubated at 25°C for 10 min, then at 50°C for 15–30 min and at 85°C for 5 min. After that, reactions were stored at −80°C.

### Quantitative Real-Time PCR and Data Analysis

Immune genes and primers used in this study are listed in Table 1. Primers were designed by Primer 3 software (http://bioinfo.ut.ee/primer3-0.4.0/), and the 3′ end of one primer was placed on intron/exon junction to eliminate any potential non-cDNA amplifications. Primers were synthesized commercially (MWG Eurofins Genomics), and real-time PCR was run using FastStart Universal SYBR Green Master Kit (ROX; Roche, Basal, Switzerland). Each qPCR reaction was 20 μl and included 10 μl FastStart Universal SYBR Green Master (ROX), 0.6 μl primers, 6.8 μl nuclease-free water, and 2 μl of cDNA. The 7,500 Real-Time PCR System (Applied Biosystems) was used to perform qPCR reactions for this study. Thermal cycler was programmed with 45 cycles of 95°C for 10 s, 95°C for 15 s, 57°C for 30 s, and 72°C for 15 s. Each sample was run triplicate.

**Table 1:** Gene names, GenBank accession numbers, and primers used in this study.

**Genes**	**Accession nos**.	**Primers**	**References**
18S ribosomal RNA	AF021880	F-GAGAAACGGCTACCACATCC	(47)
		R-GATACGCTCATTCCGATTACAG	
CD4-1	DQ435305	F-GATGTCATCATTGTAGATCTCG	This study
		R-GAGGTAGCTGGCATTTCACTCC	
CD4-2	DQ435304	F-CTGTATGTTGTATCAGCCTCTG	This study
		R-CAGTCACCTCCTTACTTTGGCTA	
CD8-α	HQ446239	F-CTACGCGGAGAGACAGTCCCAA	This study
		R-CTCACAACCCAAAAGCACATC	
CD8-β	HQ446240	F-CCATCAGGCCTGGAGAAAGCA	This study
		R-TCACCACCAGGAGTAGGACA	
IL-1β	DQ157743	F-TGATCCTTTGGCCATGAGCGGC	This study
		R-AGACATTGAAAAGCTCCTGGTC	
IL-8	AY145142	F-CAATACTTTGTGAATTTCTGC	This study
		R-TGTCCTTGGTTTCCTTCTGG	
INFγ	NC_030434	F-TTGGGCAAAGTAGAGGACACC	This study
		R-TGTTTCCACACTGCCTGTTCG	
MHC class II	AF103002	F-GACACCAGGACATGGGAGGTG	This study
		R-CGAGGAAGAAAGTTCCGGTAG	
TNFα	AJ417565	F-GCACAACAAACCAGACGAGA	This study
		R-TCGTTGTCCTCCAGTTTCAA	

For normalization, the cycle threshold (Ct) of the 18 rRNA gene was subtracted from the Ct value of the gene of interest as described in the formula: ΔCt = Ct (target gene)—Ct (reference gene) (47). Then, the ΔCt value of the control was subtracted from the ΔCt value of the treatments to calculate the ΔΔCt value for each target as described in a formula: ΔΔCt target = ΔCt target treated—ΔCt target control. Fold changes were calculated for each gene using 2^−ΔΔCt^, and used for statistical analysis to determine significant differences between treatments *Ei*Δ*evpB*, ESC-NDKL1, and *E. ictaluri* WT.

### Statistical Analyses

One-way and two-way ANOVA procedures of SAS (v 9.4, SAS Institute, Inc., Cary, NC) were used to evaluate differences in means of L/CD207^+^ cells. The level of significance for all tests was set at *P* < 0.05.

## Results

### Assessment of Immune Gene Expression and L/CD207^+^ Cell Numbers in the AK of Catfish Challenged With *E. ictaluri* LAVs and WT

**Figure 2:**
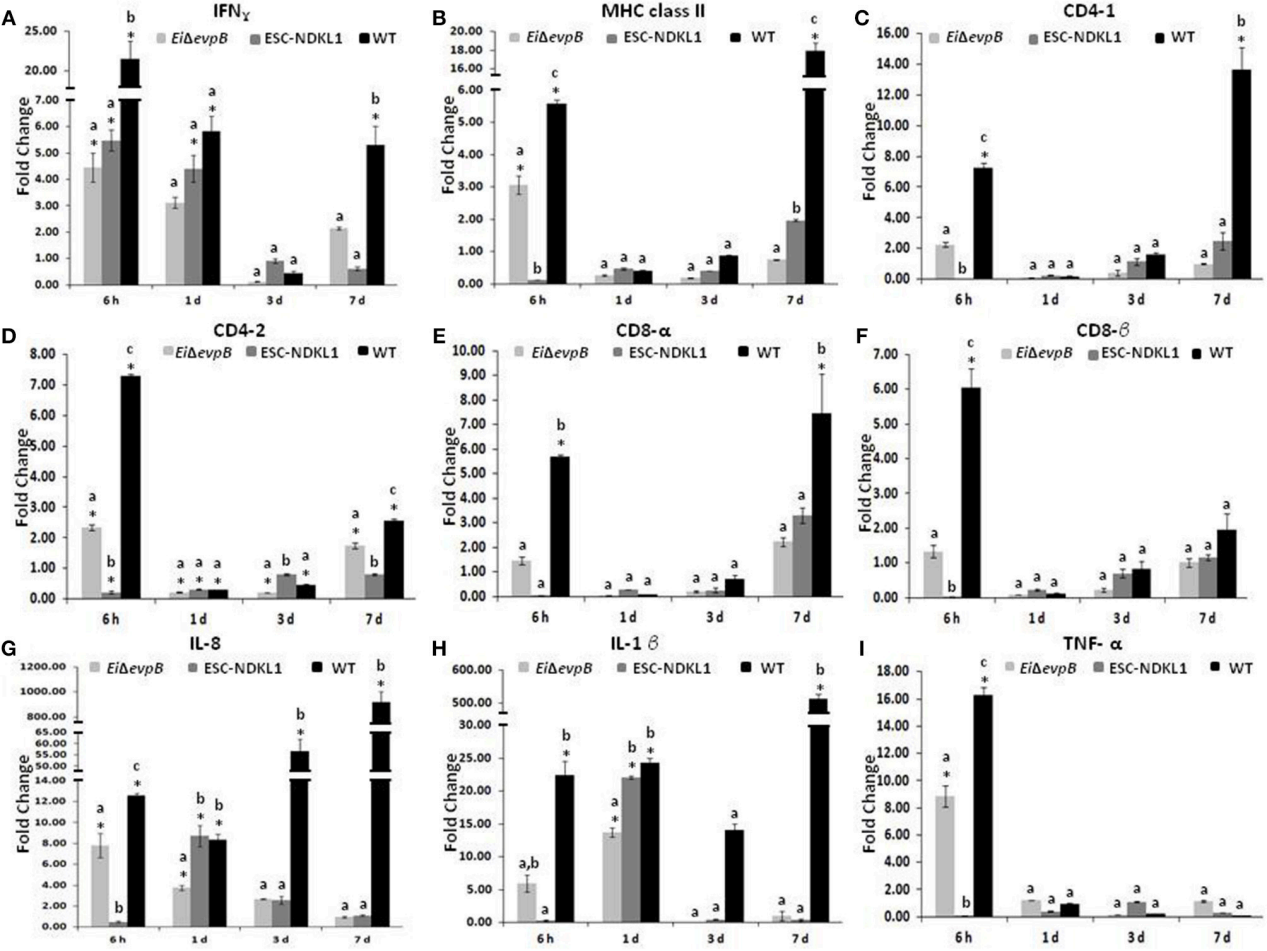
Changes in the expression of IFN-γ **(A)**, MHC class II **(B)**, CD4-1 **(C)**, CD4-2 **(D)**, CD8-α **(E)**, CD8-β **(F)**, IL-8 **(G)**, IL-1β **(H)**, and TNF-α **(I)** genes in the anterior kidney of catfish challenged with *E. ictaluri* LAV and WT strains. Data are presented as fold difference of gene expression compared with values obtained for control uninfected cells. Samples were analyzed in triplicates from a pull of AK of six fish and presented as mean ± SD. *P* < 0.05. ^*^Indicates significant differences compared to uninfected control (*p* < 0.05). ^abc^ Indicates differences within each time point (*P* < 0.05).

A significant increase in the IFN-γ gene expression was evident at 6 h, followed by a gradual decline at 7 d pc with both LAVs and the WT strain (Figure 2A). However, the IFN-γ gene expression was significantly upregulated at 7 d pc with WT only (Figure 2A). The MHC class II gene expression patterns differed between the strains with significant increases at 6 h pc with *Ei*Δ*evpB* and WT strains, followed by rapid declines at 1 and 3 d, then partial recovery after 7 d post-exposure to the ESC-NDKL1 strain (Figure 2B). In contrast, steady declines in the MHC class II gene expression were evident until a significant increase at 7 d post-exposure to the WT strain compared to the LAV-treated counterparts (Figure 2B). Importantly, ESC-NDKL1 induced significantly higher MHC class II gene expression levels compared to its LAV counterpart at 7 d pc (Figure 2B). In general, the T cell co-receptors gene expression patterns in the AK of LAVs and WT-challenged fish resembled the patterns for the MHC class II gene expression (Figures 2C–F). Pro-inflammatory chemokine and cytokine IL-8, IL-1β, and TNF-α genes were upregulated significantly in the AK at 6 h pc with *Ei*Δ*evpB* showing significant increases in IL-8 and IL-1β gene expression at 1 d pc compared to the control groups (Figures 2G–I). In contrast, IL-8 and IL-1β genes were upregulated significantly in the catfish AK at 1 d pc with ESC-NDKL1 only (Figures 2G–I). Changes in the cytokine/chemokine and lymphocyte-specific gene expression induced by WT *E. ictaluri* were evident at 6 h post-exposure, resulting in significant upregulation of most of the genes evaluated in the AK of catfish that survived infection (Figures 2A–I). However, genes that increased their expression the most were the Th1 type cytokine gene IFN-γ and pro-inflammatory cytokine/chemokine genes IL-1β, IL8, and TNF-α (Figures 2A,G–I).

**Figure 3:**
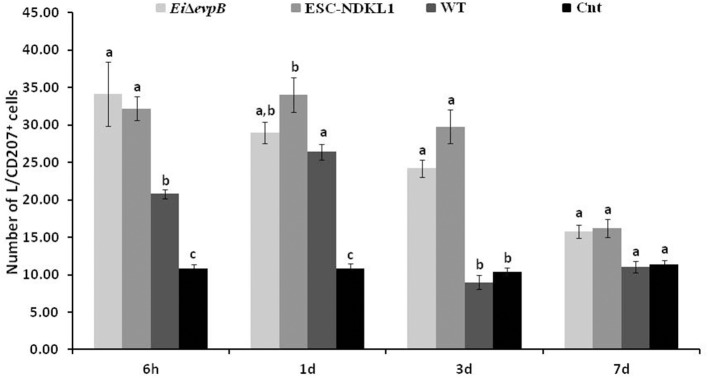
The kinetics of L/CD207^+^ cells numbers in the anterior kidney of catfish challenged with *E. ictaluri* LAV and WT strains. The (a, b, c) letters on top of bars indicate statistical group differences in L/CD207^+^ cell numbers at each time point (*P* < 0.05). The data represent the mean of cell numbers from five AKs ± SD.

The numbers of the L/CD207^+^ cells in the AK of catfish challenged with two LAV and WT strains significantly increased at 6 h pc compared to control catfish (Figure 3). Interestingly, the L/CD207^+^ cell numbers were significantly higher in the AK of catfish challenged with the LAVs compared to their counterparts challenged with the WT strain at this time point. However, there was no significant difference in the numbers of L/CD207^+^ cells between the LAVs (Figure 3).

Similar to 6 h pc, the numbers of L/CD207^+^ cells in the AK of the WT- challenged catfish were significantly higher compared to non-treated controls at 1 d pc (Figure 3). Furthermore, the numbers of L/CD207^+^ cells were significantly higher in the AK of catfish challenged with ESC-NDKL1 compared to the AK of catfish challenged with the WT strain. However, there was no significant difference between the two LAV strains (Figure 3). Interestingly, the numbers of L/CD207^+^ cells at 3 d challenge were significantly decreased in the AK of catfish challenged with the WT strain and did not differ from the uninfected control group (Figure 3). In contrast, the numbers of L/CD207^+^ cells in the AK declined compared to the 1 d challenge but were still significantly elevated in the groups challenged with both LAVs compared to their WT-treated and control counterparts (Figure 3). After 7 d post challenge, the groups exposed to LAVs showed significant decreases in the numbers of L/CD207^+^ cells compared to 3 d numbers, which did not differ in the controls (Figure 3). Overall, we documented significant declines in the numbers of LC-like cells in the AK of catfish at 3 and 7 d pc with both LAVs following similar patterns (Figure 3).

### Assessment of Immune Gene Expression and L/CD207^+^ Cell Numbers in the Spleen of Catfish Challenged With *E. ictaluri* LAVs and WT

**Figure 4:**
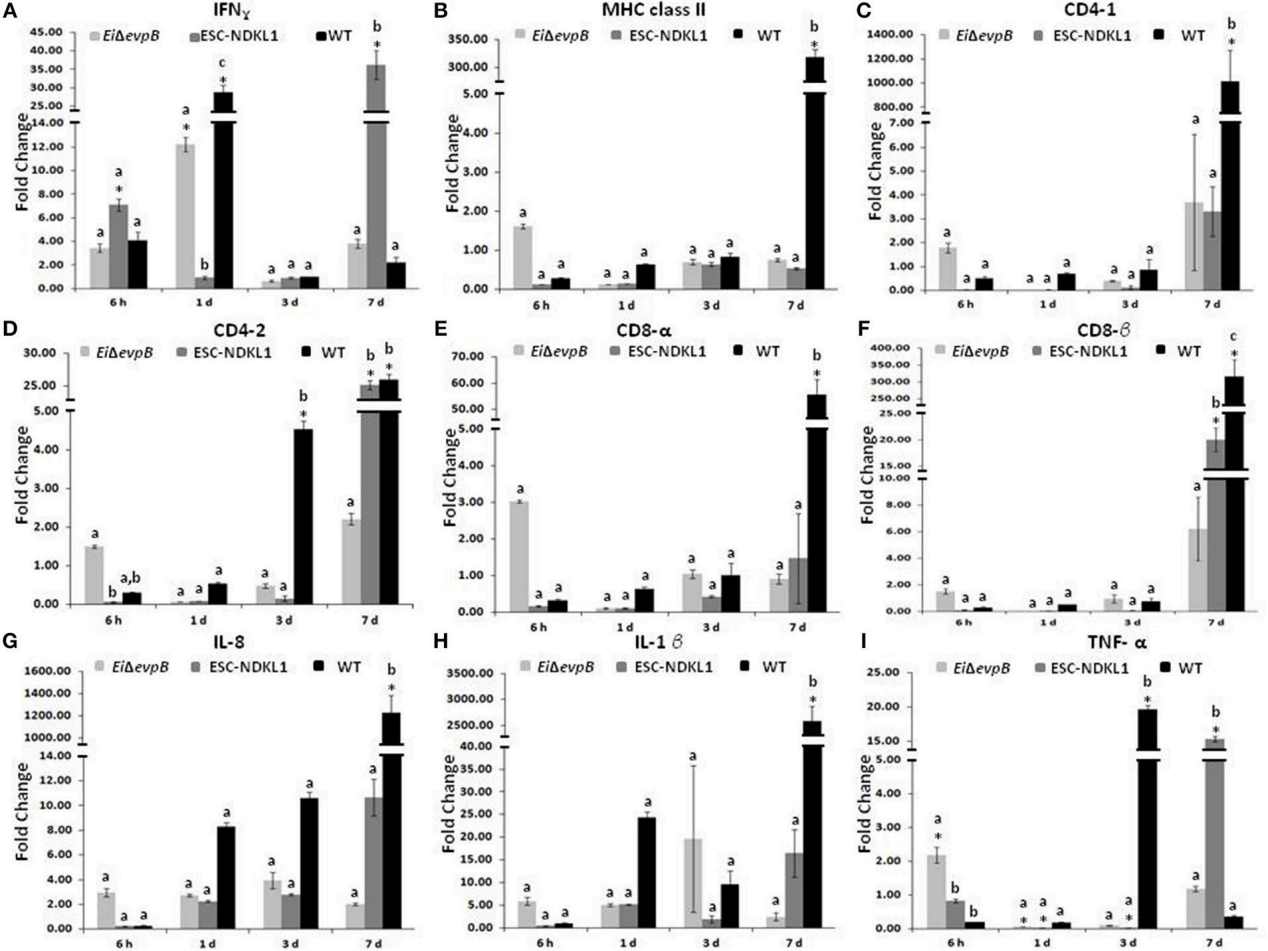
Changes in the expression of IFN-γ **(A)**, MHC class II **(B)**, CD4-1 **(C)**, CD4-2 **(D)**, CD8-α **(E)**, CD8-β **(F)**, IL-8 **(G)**, IL-1β **(H)**, and TNF-α **(I)** genes in the spleen of catfish challenged with *E. ictaluri* LAV and WT strains. Data are presented as fold difference of gene expression compared with values obtained for control uninfected cells. Samples were analyzed in triplicates from a pull of spleen of six fish and presented as mean ± SD. ^*^Indicates significant differences compared to uninfected control (*p* < 0.05). ^abc^ Indicates differences within each time point (*P* < 0.05).

Significant increases in the IFN-γ gene expression in the spleen were documented at 6 h pc with ESC-NDKL1 LAV (Figure 4A). Furthermore, significant increases in IFN-γ gene expression in the spleen were evident at 1 d and 7 d pc with *Ei*Δ*evpB* and the WT strain, and with the ESC-NDKL1 strain, respectively, compared to uninfected controls (Figure 4A). Significant increases in MHC class II gene expression at 7 d were evident in the spleen after exposure to the WT strain compared to the LAVs-exposed spleen and uninfected controls (Figure 4B). No significant changes were documented in CD4-1 gene expression following the LAVs challenge in the spleen. However, *E. ictaluri* WT induced strong upregulation of the CD4-1 T cell co-receptor gene (Figure 4C). The CD4-2 co-receptor gene was upregulated at 3 d with the WT strain only and at 7 d pc with both ESC-NDKL1 and the WT strain (Figure 4D). CD8-α gene expression levels were significantly increased in the spleen at 7 d pc with *E. ictaluri* WT (Figure 4E). However, the CD8-β gene expression patterns resembled the expression of the CD4-1 gene in both LAVs and WT treatments, with significant increases at 7 d ESC-NDKL1 and WT pcs (Figure 4F). Pro-inflammatory cytokine/chemokine IL-8 gene was significantly upregulated at 7 d pc with WT compared to the LAV-treated and untreated controls (Figure 4G). The TNF-α gene expression was significantly increased at 6 h pc with *Ei*Δ*evpB* and at 7 d pc with ESC-NDKL1, respectively (Figure 4I). No changes were found in the expression levels of IL-1β in the LAVs-challenged spleens compared to their untreated counterparts (Figure 4H). We documented significant changes in the pro-inflammatory cytokine/chemokine gene expression induced by WT *E. ictaluri* in the spleen of catfish at 3 and 7 d pc. Day 3 pc was characterized by significant increases in the TNF-α gene compared to the control and the LAVs-treated groups (Figure 4I). By day 7 of WT strain post-treatment, the gene expression pattern showed predominantly increased pro-inflammatory mediators IL-8 and IL-1β genes (Figures 4G,H).

**Figure 5:**
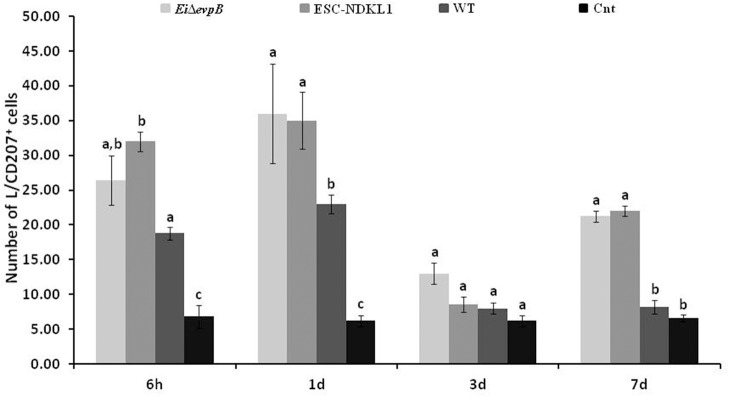
The kinetics of L/CD207^+^ cells numbers in the spleen of catfish challenged with *E. ictaluri* LAV and WT strains. The letters (a, b, c) show the significant differences between groups at each time point (*P* < 0.05). The data represented the mean of five fish ± SD.

We evaluated the L/CD207^+^ cell numbers in the spleen of catfish challenged with *E. ictaluri* LAV and WT strains (Figure 5). The L/CD207^+^ cell numbers in the spleen at 6 h showed significant treatment-related differences but were comparable to the LC-like cell numbers at 1 d pc (Figure 5). Namely, L/CD207^+^ cell numbers significantly increased in the spleen of catfish challenged with two LAVs and WT strains compared to non-vaccinated catfish, whereas there was no significant difference between *Ei*Δ*evpB* and ESC-NDKL1 strains (Figure 5).

As expected, the numbers of L/CD207^+^ cells in the spleen dramatically decreased in all groups at 3 d pc with no significant differences between treated and control fish (Figure 5). However, both LAV strains followed a similar pattern in the L/CD207^+^ cell numbers, which was different from the WT-treated and control groups showing a significant increase in the L/CD207^+^ cell numbers at 7 d pc (Figure 5). Overall, we showed that the kinetics of the L/CD207^+^ cell numbers in the *Ei*Δ*evpB*- treated groups were similar to the kinetics of their ESC-NDKL1-treated counterparts. Furthermore, significant drops in the LC-like numbers occurred at 3 d pc in LAV and WT strain-challenged spleen followed by dramatic increases in LAVs-treated groups at 7 d post-exposure.

### Assessment of L/CD207^+^ Cell Numbers in the Gills of Catfish Challenged With *E. ictaluri* LAVs and WT

**Figure 6:**
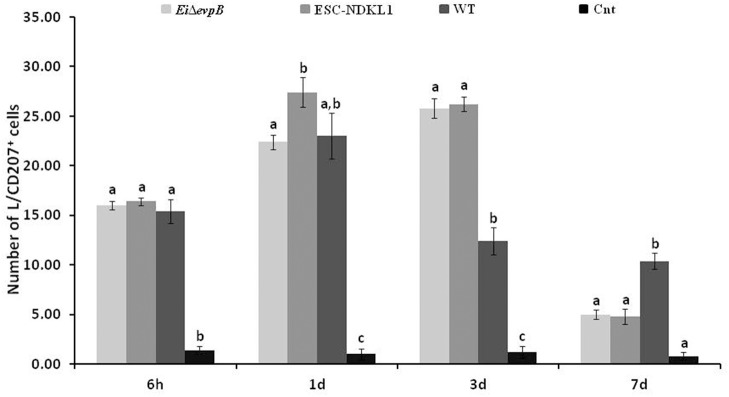
The kinetics of L/CD207^+^ cells numbers in the gills of catfish challenged with *E. ictaluri* LAV and WT strains. Different letters (a, b, and c) show the significant differences between groups at each time point (*P* < 0.05). The data represent the mean of cell numbers in five fish gills ± SD.

In this study, we evaluated L/CD207^+^ cell numbers in the gills of catfish challenged with *E. ictaluri* LAVs and the WT strain at different time points (Figure 6). After 6 h pc infection, L/CD207^+^ cell numbers significantly increased in the gills of catfish vaccinated with both the LAV and WT strains compared to non-vaccinated fish, and there was no significant difference in L/CD207^+^ cell numbers between LAVs and WT strains (Figure 6). The numbers of L/CD207^+^ cells in the gills of all treatment groups continued to increase at 1 d of pc, and there was a significant difference between *Ei*Δ*evpB* and ESC-NDKL1 strains but not compared to the WT strain (Figure 6). No significant shifts were documented in L/CD207^+^ cell numbers in the gills of catfish vaccinated with both LAVs at 3 d pc. However, L/CD207^+^ cell numbers dramatically decreased in the gills of fish challenged with WT but were still significantly higher than in non-challenged fish. On the other hand, after 7 d pc, L/CD207^+^ cell numbers significantly decreased in the gills of fish vaccinated with both LAVs and were not different from non-vaccinated fish control group (Figure 6). The numbers of L/CD207^+^ cells were significantly higher in catfish vaccinated with the WT strain compared to LAVs-treated and control groups (Figure 6). In summary, the most dramatic changes in the numbers of LC-like cells in the gills were evident as increases at 3 d pc and decreases at 7 d pc with LAVs compared to the WT strain treated and control groups, respectively (Figure 6).

## Discussion

The critical function of DCs is to bridge innate antigen recognition and adaptive immune responses. Recent studies showed that DCs in teleost fish have similar morphology and function as mammalian DCs (26, 28). Previously, our group showed the presence of DCs with remarkable similarities to human LCs in the spleen, anterior and posterior kidneys, and gills of channel catfish (40).

Due to the lack of information available on the mechanisms of immune responses to efficacious *E. ictaluri* LAVs, we assessed expression of the genes related to innate and specific antigen presentation and the genes that promote inflammation in the persistently infected immunocompetent tissues of catfish. Due to the lack of information available on the morphological and functional markers of catfish DCs, we applied the IHC approach described previously (40) to evaluate the LC-like expression patterns in the immune-related tissues of catfish challenged with two LAVs and WT *E. ictaluri* strains to underscore their immune effector mechanisms.

Our data suggest that rapid bacterial colonization in the AK of channel catfish challenged with *E. ictaluri* LAVs and WT at the early stage is combined with elevated IFN-γ gene and increased numbers of L/CD207^+^ cells. This observation can be explained by the development of innate immune responses at the site of infection, and two LAVs were more efficient at inducing DC numbers compared to their WT counterpart. Importantly, the increased numbers of L/CD207^+^ cells in combination with elevated IFN-γ gene expression at the early stages of exposure, followed by significant decreases in the DC-like cell numbers and relatively low expressed IFN-γ gene at 7 d post-exposure in catfish vaccinated with both LAVs, suggest the involvement of LC in the initiation of innate immune responses and their migration/maturation from the AK to the site of infection, the gills, after 3 day pc.

The ESC-NDKL1-induced IFN-γ gene expression patterns in general resembled the patterns induced by the *Ei*Δ*evpB* strain in the AK of catfish. However, differences regarding the MHC class II and T cell-related co-receptor gene expression patterns between two LAVs and the WT strain could be due to a different framework of initiation of innate and adaptive immune responses by *Ei*Δ*evpB* compared to the ESC-NDKL1 strain, in particular, earlier onset of innate immune mechanisms. Our previous reports showed vaccine strain-dependent differences in fish mortality rates, humoral immune responses, uptake, and bacterial killing properties of peritoneal macrophages (14, 16). In contrast to both LAVs, the WT strain significantly increased the immune gene expression levels of MHC class II, CD4-1, and CD8α in the AK of catfish fingerlings, which could be explained by the overall significantly higher bacterial load of *E. ictaluri* WT compared to the mutant strains, including both LAVs (48), Ibrahim et al., unpublished observation. Although we documented increases in the activity of the genes encoding pro-inflammatory cytokines/chemokines in the AK of catfish vaccinated with both LAVs, they were not as dramatic and consistent as their counterparts in the WT *E. ictaluri*- infected fish that caused high mortality rates in fish (15, 16). In addition, our data on significant increases in the numbers of L/CD207^+^ cells at 6 h and 1 d pc with *E. ictaluri* strains in the AK of catfish agree with and contribute to the previous report that DC-SCRIPT, a barramundi fish DC specific marker, in combination with MHCII expression, was significantly upregulated in the AK of barramundi at both 6 h and 1 d post-injection with peptidoglycan (PTG) and lipopolysaccharide (LPS), but relative expression of DC-SCRIPT was low at 3 and 7 d (30).

Importantly, significant increases in the expression of Th1 type polarization-related genes of adaptive immune responses were documented in the spleen at 7 d post-treatment with ESC-NDKL1 only. Namely, the IFN-γ gene was upregulated after exposure to the ESC-NDKL1 strain at 7 d post-exposure. Also, significant increases in the gene expression of T cell co-receptors, CD4-2 and CD8-β were evident in the spleen at 7 d post-exposure to the ESC-NDKL1 strain. Although as bacterial loads decreased (22), the dominant environment in the spleen of catfish survivors was inflammatory with dramatically overexpressed IL-1β and IL-8 mediators of inflammation at 7 d post-exposure to *E. ictaluri* WT. Interestingly, catfish fingerlings that survived the WT strain challenge 7 d pc showed significantly increased multiple adaptive immunity-related genes such as CD4 and CD8 T cell co-receptors and MHC class II, suggesting initiation of antibacterial immune responses under inflammatory conditions. Significantly elevated ESC-NDKL1-induced TNF-α gene expression levels in the spleen of catfish at 7 d pc could explain the higher mortality rates in catfish fingerlings challenged with the ESC-NDKL1 strain compared to its LAV counterpart (16).

Our data on dramatic increases in the numbers of L/CD207^+^ cells in the spleen at 6 h pc, possibly via cytokine signaling cascades involving DCs, are due to the two LAVs being more efficient at inducing innate immune responses compared to their WT counterpart. The significant drops in the numbers of L/CD207^+^ cells in the spleen at 3 d pc suggest possible migration of these cells from the spleen to the site of infection followed by remarkable increases in LC-like numbers in the spleens of the vaccinated catfish after 7 d of the treatment. The LAVs induced increases in the spleen but not in the AK after 7 days of challenge, suggesting the migration of LC-like cells back to the spleen to present the pathogen-derived antigen to specific T cells, thus initiating adaptive immune responses. Similarly to our findings, the study with barramundi reported that the expression of DC-SCRIPT was higher in the spleen at 6 h post-injection of LPS and PTG, but its expression level decreased in the spleen after 1 d, and DC-SCRIPT expression was again elevated at 7 d (30). The MHC class II gene expression patterns did not always correlate with the kinetics of the L/CD207^+^ cell numbers. However, in the spleen, their significant increases were evident at 3 and 7 d compared to their counterparts at 6 h and 1 d post-treatment with both LAVs. The MHC class II molecules, unlike the DC-SCRIPT assessed in barramundi, are not limited to the pan-DC populations only but also expressed in other professional APCs, B cells and monocytes/macrophages (49–52).

Gills are one of the potential routes of entry for *E. ictaluri* into the channel catfish host (53). Our data on the significant spikes in the numbers of L/CD207^+^ cells in the gills in all fish at 6 h, 1 and 3 d pc followed by decreases after 7 days could be interpreted as possible antigen recognition and capture by immature DCs. Our data support the previous observation that DC-like cells have been described in the gills of Chinook salmon heavily infected with *L. salmonae* (39). We expanded this earlier report by showing the kinetics of L/CD207^+^ cells expression patterns in catfish gills at different time points of exposure to the LAVs and WT *E. ictaluri* strains. Our data suggest that L/CD207^+^ cells in the gills may recognize and capture antigens at 6 h, 1 d, and 3 d pc, and after 7 d, L/CD207^+^ cells in the gills of catfish vaccinated with two LAV strains have migrated to the secondary lymphoid tissue in the spleen for antigen presentation. However, the APC function of L/CD207^+^ cells and, in particular, their migration to the spleen in the gills of catfish infected with WT strain was impaired.

In conclusion, followed by our previous observations on the vaccine strain-dependent differences in bacterial tissue persistence, fish mortality rates, humoral immune responses, uptake, and bacterial killing properties of macrophages (14, 16) here we report the differential framework of the initiation of innate and adaptive immune responses between two LAV and WT strains.

## Author Contributions

LP and AK conceived and designed the experiments. LP and AK provided the original idea of the study. AOK, HoA, and HaA performed the experiments. LP, AK, and WB contributed reagents, materials, tools. AOK wrote the first draft of the manuscript and was involved in all aspects of the study. All authors were involved in critical interpretation of the data, manuscript revision, and final version approval.

### Conflict of Interest Statement

The authors declare that the research was conducted in the absence of any commercial or financial relationships that could be construed as a potential conflict of interest.
