# Supplementary material for: Assessment of the Live Attenuated and Wild-Type Edwardsiella ictaluri-Induced Immune Gene Expression and Langerhans-Like Cell Profiles in the Immune-Related Organs of Catfish
Source: Front Immunol. 2019 Mar 6;10:392. doi: 10.3389/fimmu.2019.00392 (PMC6414466; doi:10.3389/fimmu.2019.00392)
Supplement: Supplementary file 1 [file Data_Sheet_1.PDF]

Supplementary Figures

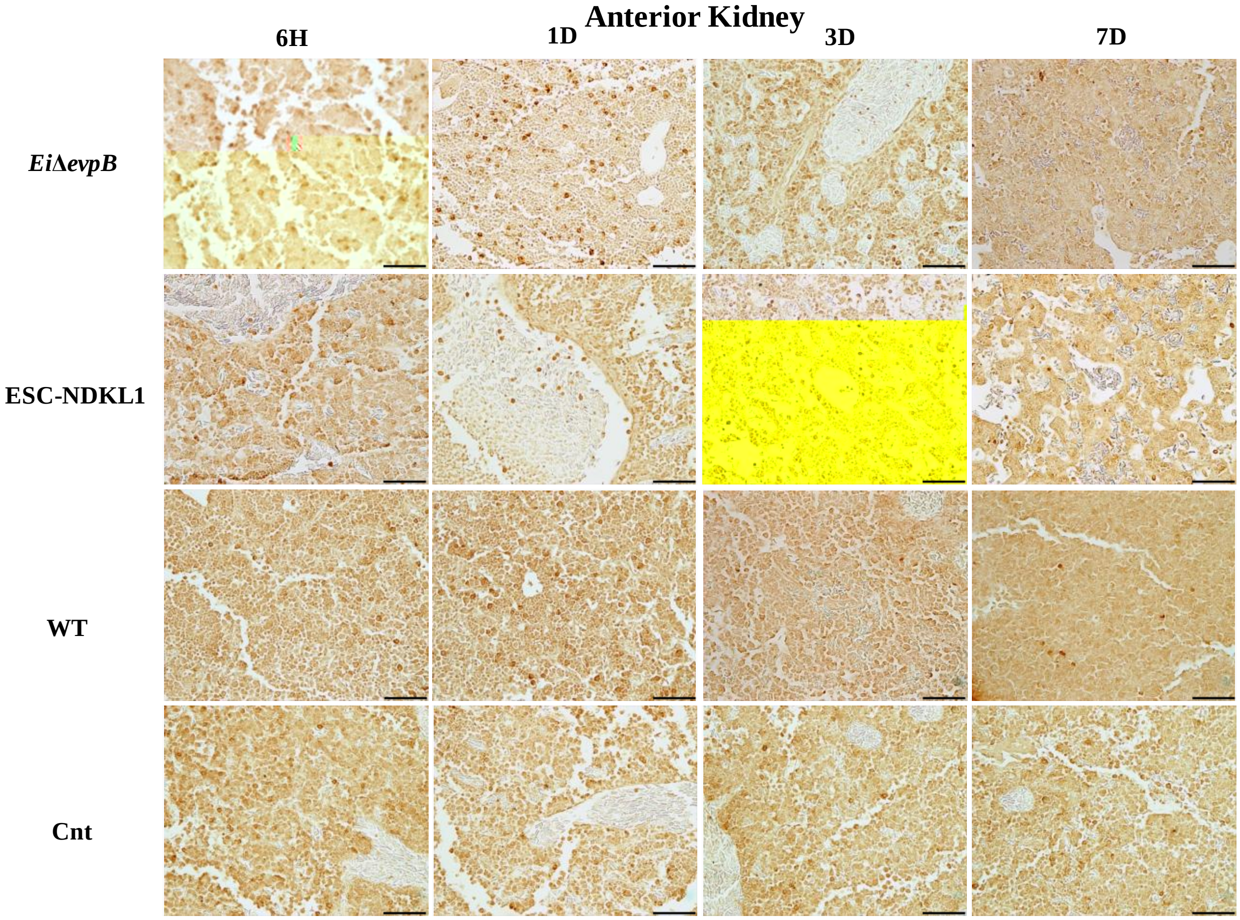

Supplementary Figure 1. Numbers of L/CD207^{+} cells in the AK of channel catfish challenged with *E. ictaluri* LAV and WT strains at 6 h, 1 d, 3 d, and 7 d post-challenge. Photomicrographs (400 x magnification, scale bar 50 μm).

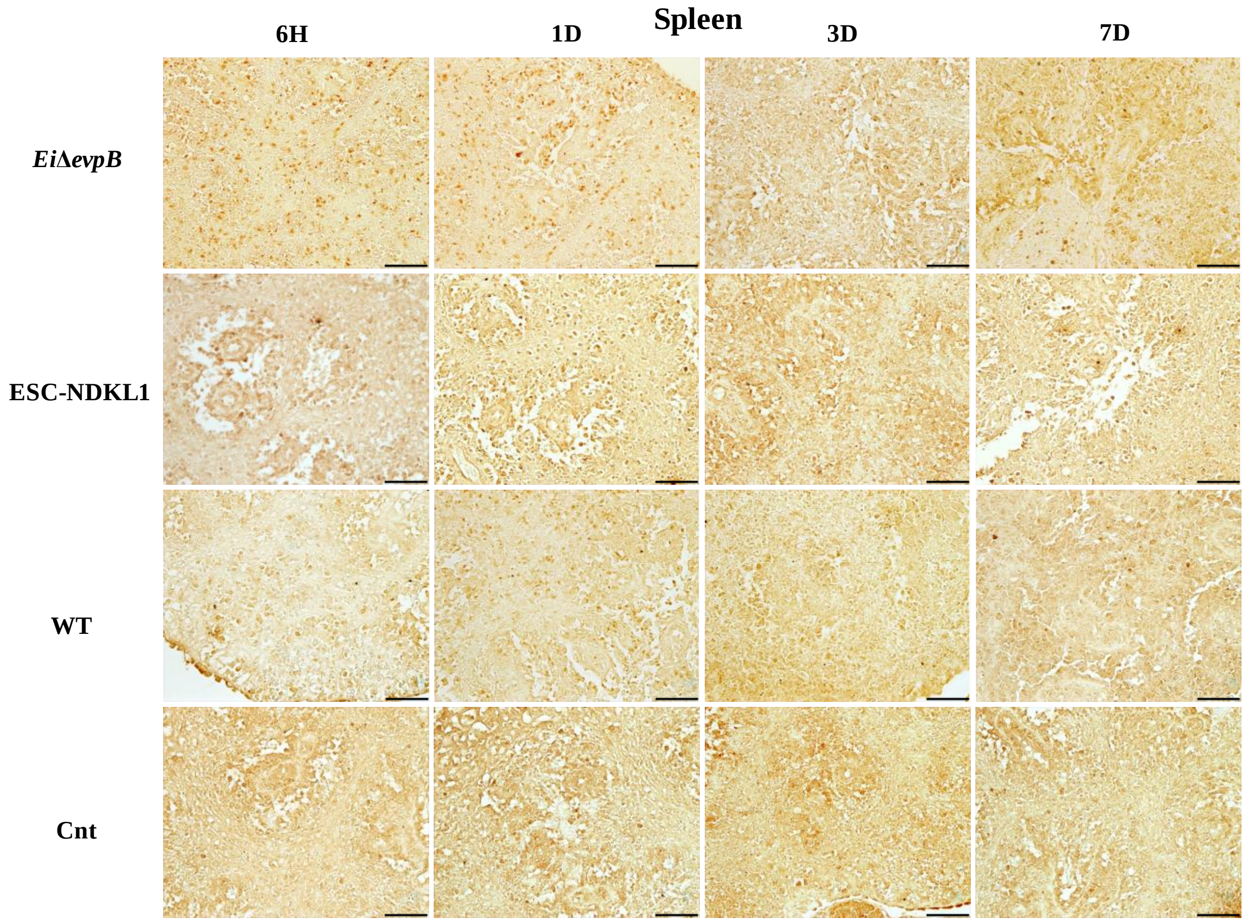

Supplementary Figure 2. Numbers of L/CD207^{+} cells in the spleen of channel catfish challenged with *E. ictaluri* LAV and WT strains at 6 h, 1 d, 3 d, and 7 d post-challenge. Photomicrographs (400 x magnification, scale bar 50 μm).

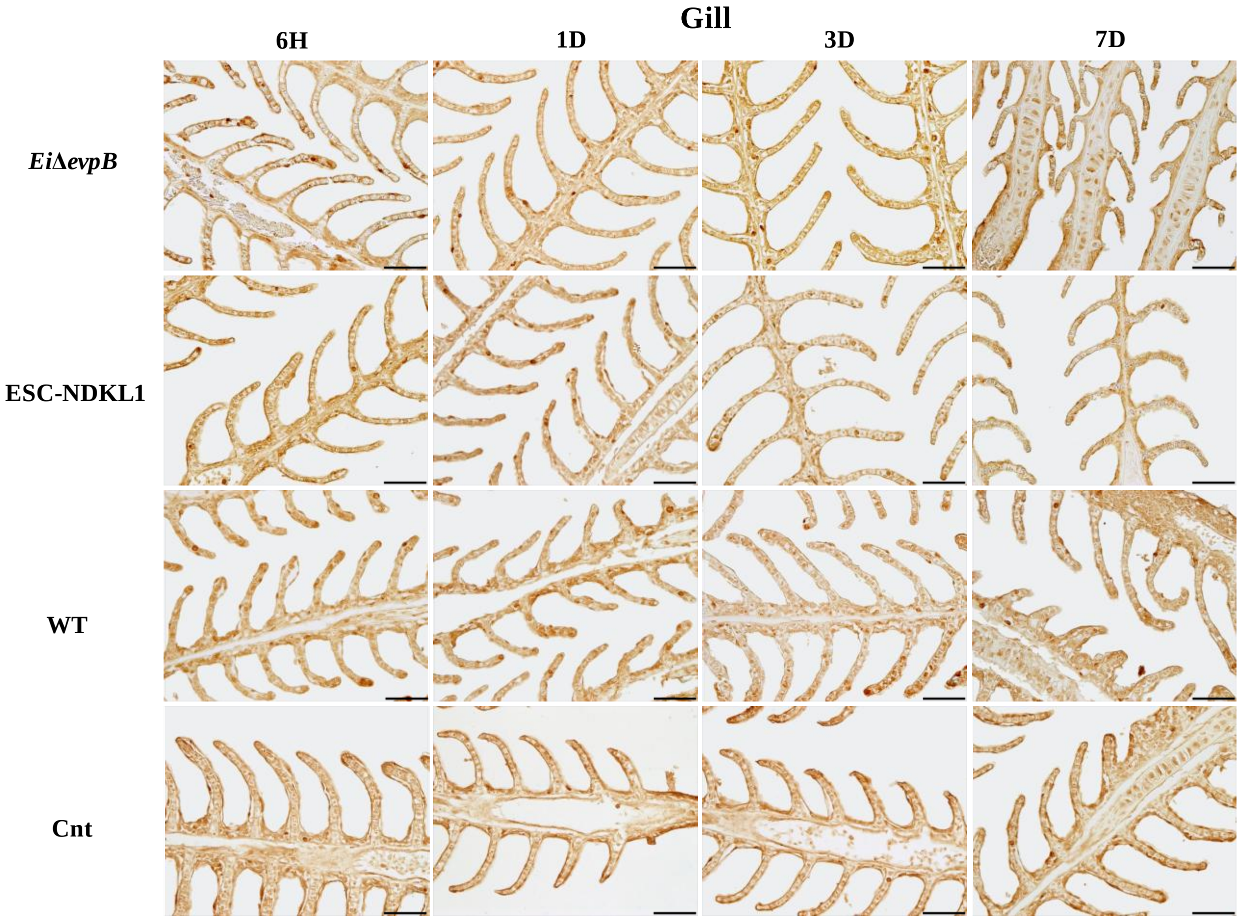

Supplementary Figure 3. Numbers of L/CD207^{+} cells in the gill of channel catfish challenged with *E. ictaluri* LAV and WT strains at 6 h, 1 d, 3 d, and 7 d post-challenge. Photomicrographs (400 x magnification, scale bar 50 μm).
